# 

**Published:** 1916-04-29

**Authors:** 


### CONTENTS.

| | PAGE |
|---|---|
| **The Civil Hospitals and the War Office** ... | 93 |
| **"Riders" by Coroners' Juries** ... ... ... | 94 |
| **Hospital and Institutional News** ... ... ... | 95 |
| Our Special Number—Honours for the Wittenberg Doctors—Typhus in Other German Prisons—St. George's Hospital Secretaryship—Invalid War Prisoners in Switzerland—The Schedule of Diseases—The Cheerful Wounded in France—Our Oversea Soldiers—A Unanimous Verdict—Bring the Men Together—A Canadian Hospital for French Wounded—A Motto for Hospital Editors—A Sonnet to a Nurse—Should Hospitals Keep Fowls?—A Named Ward at Huddersfield—Notifiable Disease in 1915—Doctors' Fees and Motor-Cars—The Future of the Fresh Air Fund—The Centenary of the Royal Waterloo Hospital for Children and Women—A Stockport Appointment—A Consulting Staff for the Leeds Union Infirmary—The Imperfections of Human Nature—A War-time Amendment of the Poor-Law Superannuation Act—Children's Infirmary, Cleveland Street, W.—A Handbook of Tuberculosis Schemes—The Supply of Dutiable Commodities to Hospitals—A Place of "Safety"!!!—This Week's Drug Market. | |
| **Cerebro-Spinal Meningitis** ... ... ... ... | 101 |
| Some of Its Problems. | |
| **London and Counties Medical Protection Society** ... ... ... ... ... ... | 102 |
| **The School Doctor** ... ... ... ... ... | 103 |
| Ideals for the Future. | |
| **From Across the Seas** ... ... ... ... | 104 |
| Where Babies Thrive—Antipodean Salaries—The Surgeon's Pledge. | |
| **National Insurance Act Developments**... ... | 105 |
| The Inquiry and After. | |
| **Parliament and the Profession** ... ... ... | 106 |
| **The College of Nursing, Limited** ... ... | 107 |
| By the Honourable Arthur Stanley, M.P., M.V.O., Chairman of the Council. | |
| **The Matrons' and Sisters' Department** ... | 109 |
| The Matrons' Experiences. | |
| Round the Hospitals. | |
| Mr. Stanley's Letter—Liverpool Notes—Nurses' Uniform—Rising Price of Food—The Blind Girl—Plymouth Madrigal Society—"The Outlook for the Matron"—Prize for Best Story. | |
| **A Garden Colony for Consumptives** ... ... | 111 |
| **The Editor's Letter-Box** ... ... ... ... | 112 |
| With the Wounded: The Growing and Propagation of Bulbs—St. George's Hospital—The College of Nursing Meeting. | |
| **Personalia**... ... ... ... ... ... ... ... | 112 |
| **Textile Supplies for Hospitals** ... ... ... | 113 |
| X.—Sheets, Towels, and Table Linens. | |
| **Passing Events and Topics** ... ... ... ... | iv |
| **Matrons' and Sisters' Appointments** ... ... | iv |
| **The Institutional Worker** (Suppl.) | 1 |
| Organising a Newspaper Collection. | |
| A Self-Adjustable Lid. | |
| Splint for Colles' Fracture of the Arm. | |

